# pH-Responsive Micelle-Based Cytoplasmic Delivery System for Induction of Cellular Immunity

**DOI:** 10.3390/vaccines5040041

**Published:** 2017-11-04

**Authors:** Eiji Yuba, Naoki Sakaguchi, Yuhei Kanda, Maiko Miyazaki, Kazunori Koiwai

**Affiliations:** 1 Department of Applied Chemistry, Graduate School of Engineering, Osaka Prefecture University, 1–1 Gakuen–cho, Naka–ku, Sakai, Osaka 5998531, Japan; kndyh.june8@gmail.com (Y.K.); su108056@edu.osakafu-u.ac.jp (M.M.); 2 Terumo Corp., Ashigarakami–gun, Kanagawa 2590151, Japan; Naoki_Sakaguchi@terumo.co.jp (N.S.); tgwmt240@jcom.zaq.ne.jp (K.K.)

**Keywords:** micelle, pH-responsive, deoxycholic acid, dendritic cell, cellular immunity

## Abstract

(1) Background: Cytoplasmic delivery of antigens is crucial for the induction of cellular immunity, which is an important immune response for the treatment of cancer and infectious diseases. To date, fusogenic protein-incorporated liposomes and pH-responsive polymer-modified liposomes have been used to achieve cytoplasmic delivery of antigen via membrane rupture or fusion with endosomes. However, a more versatile cytoplasmic delivery system is desired for practical use. For this study, we developed pH-responsive micelles composed of dilauroyl phosphatidylcholine (DLPC) and deoxycholic acid and investigated their cytoplasmic delivery performance and immunity-inducing capability. (2) Methods: Interaction of micelles with fluorescence dye-loaded liposomes, intracellular distribution of micelles, and antigenic proteins were observed. Finally, antigen-specific cellular immune response was evaluated in vivo using ELIspot assay. (3) Results: Micelles induced leakage of contents from liposomes via lipid mixing at low pH. Micelles were taken up by dendritic cells mainly via macropinocytosis and delivered ovalbumin (OVA) into the cytosol. After intradermal injection of micelles and OVA, OVA-specific cellular immunity was induced in the spleen. (4) Conclusions: pH-responsive micelles composed of DLPC and deoxycholic acid are promising as enhancers of cytosol delivery of antigens and the induction capability of cellular immunity for the treatment of cancer immunotherapy and infectious diseases.

## 1. Introduction

Cell-mediated immune response (cellular immunity) plays a crucially important role in the treatment of chronic infectious disease, elimination of virus-infected cells, prophylactic vaccine, and cancer immunotherapy because cytotoxic T lymphocytes (CTLs) can attack tumor cells and virus-infected cells directly [1,2,3,4]. For induction of CTL-based cellular immunity, delivery of antigens into the cytosol of antigen-presenting cells, such as macrophages or dendritic cells (DCs), is necessary, which leads to the processing of antigens via proteasome, antigen-loading on major histocompatibility complex (MHC) class I, and antigen presentation to CD8-positive T lymphocytes [5,6]. In general, after an exogenous antigen is taken up by antigen-presenting cells via endocytosis it is processed in endo/lysosomes, which leads to MHC class II-mediated antigen presentation to CD4-positive T lymphocytes [5,6]. Therefore, promotion of exogenous antigen transfer into cytosol using endosomolytic reagents or intracellular delivery carriers is necessary to achieve antigen-specific cellular immunity. This process is known as “cross-presentation” [7]. For instance, endosomolytic reagents, such as chloroquine, promote the transfer of antigen into cytosol of dendritic cells in vitro, probably because of its pH-buffering effect in endo/lysosomes, which induce cross-presentation [8,9]. However, low-molecular-weight reagents might be diluted to less than active concentration in the body and might have difficulty achieving promotion of cytosolic transfer of antigens under in vivo conditions.

An antigen delivery carrier is another candidate to promote cross-presentation, even under in vivo conditions. Typically, fusogenic protein-incorporated liposomes such as Virosome or Sendai virus fusogenic protein-loaded liposomes are used for the cytoplasmic delivery of antigens [10,11]. These antigen-loaded liposomes can deliver antigens into cytosol via fusion with endosomal membranes or plasma membranes. Synthetic endosomolytic materials are also used as cytoplasmic delivery systems. Poly(carboxylic acid)s are well-studied pH-responsive endosomolytic materials because poly(carboxylic acid)s change their structure from a coil to a globule after protonation of carboxyl groups and interact with lipid membranes via hydrogen bond formation with phosphate groups on membranes and hydrophobic interactions [12,13,14]. Poly(acrylic acid) derivatives, such as poly(ethyl acrylic acid) and poly(propylacrylic acid), achieve membrane lysis at acidic pH. Actually, conjugation of poly(propylacrylic acid) to antigenic proteins promotes cross-presentation in vitro and the induction of antigen-specific cellular immune response in vivo [15,16]. We also developed pH-responsive polymers using carboxylated polyallylamines, polyglycidols, or polysaccharides [17,18,19,20,21,22,23]. Particularly, liposomes modified with carboxylated polyglycidols or carboxylated polysaccharides having ether oxygen atoms in their backbone exhibited membrane fusion with the target membrane under weakly-acidic pH. Model antigenic protein, ovalbumin (OVA), was delivered into the cytosol of DCs and OVA-specific cellular immunity was induced, resulting in tumor regression in tumor-bearing mice [20,21,24]. Consequently, pH-responsive polymer-modified liposomes are effective for the induction of cellular immunity by membrane fusion responding to weakly-acidic pH in endosomes of DCs. However, precise quality control of responsiveness and pH where membrane fusion is induced might be difficult because of the polydispersity of synthetic polymers. Therefore, a more versatile cytoplasmic delivery carrier is desired with simple composition from the viewpoint of practical or clinical use.

**Figure 1:**
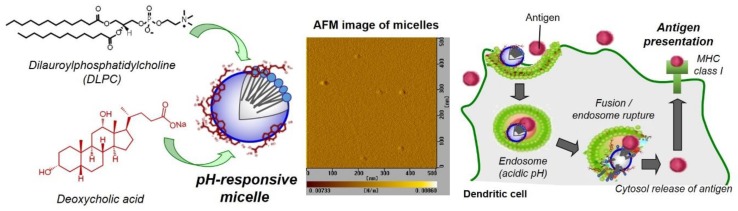
Design of pH-responsive micelles composed of DLPC and deoxycholic acid for antigen delivery into cytosol of dendritic cells and induction of antigen presentation via MHC class I molecules (cross-presentation), which leads to antigen-specific cellular immunity. An atomic force microscope (AFM) image for DLPC/deoyxcholic acid micelles is also shown.

Recently, we developed a pH-responsive micelle-based intracellular delivery system using dilauroyl phosphatidylcholine (DLPC) and deoxycholic acid (Figure 1). The molecular assembly behavior of DLPC and deoxycholic acid mixture were evaluated precisely using X-ray scattering analysis. They formed micelles with average size of 12 nm and negative zeta potential [25]. The DLPC/deoxycholic acid micelles showed membrane disruptive activity under weakly-acidic pH [25]. At acidic pH, protonation of carboxyl group in deoxycholic acid changed the micelle surface properties from hydrophilic to hydrophobic, which promotes the interaction of micelle with lipid membrane. These micelles also promoted the transfer of co-existing OVA molecules into cytosol of macrophages [25]. Considering the cytoplasmic delivery performance of DLPC/deoxycholic acid micelles, in this study, the pH-responsive membrane disruptive mechanism of DLPC/deoxycholic acid micelles was further evaluated using a fluorescence resonance energy transfer technique. In addition, the interaction of DLPC/deoxycholic acid micelles with DCs, which are professional antigen-presenting cells and play a crucial role for induction of CTLs, were examined. Furthermore, induction of cellular immunity in vivo by these micelles was investigated.

## 2. Materials and Methods

### 2.1. Materials

Egg yolk phosphatidylcholine (EYPC), l-dioleoyl phosphatidylethanolamine (DOPE), and 1,2-dilauroyl-*sn*-glycero-3-phosphocholine (DLPC) were purchased from NOF Corp. (Tokyo, Japan). *N*-(7-Nitrobenz-2-oxa-1,3-diazol-4-yl) dioleoyl phosphatidylethanolamine (NBD-PE), lissamine rhodamine B-sulfonyl phosphatidylethanolamine (Rh-PE) and cholesteryl hemisuccinate (CHEMS) were purchased from Avanti Polar Lipids (Birmingham, AL, USA). Deoxycholic acid sodium salt monohydrate, oleic acid, and chlorpromazine hydrochloride were purchased from Nacalai Tesque Inc. (Kyoto, Japan). Pyranine was purchased from Tokyo Chemical Industries Ltd. (Tokyo, Japan). *p*-Xylene-*bis*-pyridinium bromide (DPX), filipin complex from *Streptomyces filipinensis*, OVA, and Triton X-100 were purchased from Sigma (St. Louis, MO, USA). FITC-labeled ovalbumin (FITC-OVA) were prepared as reported previously [24]. 2-Morpholinoethanesulfomic acid sodium salt (MES) was purchased from Merck (Darmstadt, Germany). Amiloride hydrochloride dihydrate was obtained from LKT Laboratories Inc. (St. Paul, MN, USA). LysoTracker Red was purchased from Lonza (Walkersville, MD, USA). CpG-ODN (ODN-2395) was obtained from Invitrogen Corp. (San Diego, CA, USA). OVA CTL epitope peptide (SIINFEKL) was obtained from PH Japan Co., Ltd. (Hiroshima, Japan).

### 2.2. Preparation of Micelles or Liposomes

A thin membrane of phospholipid (1 μmol, EYPC or DLPC) and/or deoxycholic acid (1.6 μmol) was dispersed in 1 mL of Dulbecco’s phosphate-buffered saline (dPBS) or MES buffer (25 mM MES and 125 mM NaCl, pH 7.4) using a bath-type sonicator for 2 min (final lipid concentration, 1 μmol/mL). pH-sensitive liposomes composed of DOPE/oleic acid or DOPE/CHEMS were prepared according to earlier reports of the literature [26,27]. Briefly, a thin membrane of DOPE (1 μmol) and CHEMS (0.67 μmol) or oleic acid (0.43 μmol) was dispersed in 1 mL of acetate buffer (25 mM acetate and 125 mM NaCl, pH 7.4) using a bath-type sonicator for 2 min.

Pyranine-loaded liposomes were prepared from thin membrane of EYPC dispersed by aqueous 35 mM pyranine, 50 mM DPX, and 25 mM MES solution (pH 7.4). The mixture was sonicated for 2 min using a bath-type sonicator. The liposome suspension was extruded through a polycarbonate membrane with pore size of 100 nm. The liposome suspension was applied to a G100 column to remove free pyranine from the pyranine-loaded liposomes.

For lipid mixing assay, EYPC liposomes containing NBD-PE and Rh-PE were prepared using the method described above with the membrane composed of EYPC, NBD-PE, and Rh-PE (98.8:0.6:0.6, mol/mol/mol).

For evaluation of cellular association of micelles, DLPC/deoxycholic acid micelles containing Rh-PE were prepared as described above except that mixtures of lipids containing Rh-PE (0.6 mol %) were dispersed in dPBS.

For intracellular distribution of micelles or liposomes, liposomes or micelles containing NBD-PE and Rh-PE were prepared as described above except that mixtures of lipids containing NBD-PE and Rh-PE (each 0.6 mol %) was dispersed in dPBS.

All formulations used in this study are summarized in Supplementary Materials Table S1.

### 2.3. Atomic Force Microscopy (AFM)

AFM measurements were taken using a probe station and a unit system of the scanning probe microscopy system (SPI3800, SPA400; Seiko Instruments Inc., Chiba, Japan). The silicon cantilever (SI-DF40; Seiko Instruments Inc., Chiba, Japan) had a spring constant of 16 N/m. The micelle suspension was applied to freshly cleaved mica and incubated on the mica for 30 min. Measurements were taken in dynamic force mode (noncontact mode).

### 2.4. Cell Culture

DC2.4 cells, an immature murine DC line, were provided by Dr. K. L. Rock (Harvard Medical School, Boston, MA, USA). They were grown in RPMI 1640 supplemented with 10% Fetal bovine serum (FBS) (MP Biomedicals Inc., Santa Ana, CA, USA), 2 mM l-glutamine, 100 μM non-essential amino acids (Gibco, Inc., Billings, MT, USA), 50 μM 2-mercaptoethanol, and antibiotics at 37 °C [28].

### 2.5. Interaction of Micelles with Liposomes

Pyranine-loaded liposomes (final lipid concentration; 6.7 nmol/mL) were mixed with equal amounts of micelles or liposomes in MES buffer of varying pH at 37 °C for 90 min. The fluorescence intensity at 512 nm with excitation at 416 nm was followed using a spectrofluorometer (FP-6500; Jasco Corp., Tokyo, Japan). The percentage of leakage of pyranine from liposomes was defined as:

Leakage (%) = (*L*_t_ − *L*_i_)/(*L*_f_ − *L*_i_) × 100

where *L*_i_ and *L*_t_ respectively represent the initial and intermediary fluorescence intensities. *L*_f_ is the fluorescence intensity after addition of Triton X-100 (final concentration: 0.1%) to achieve complete membrane disruption.

### 2.6. Lipid Mixing Assay

Lipid mixing behavior between EYPC liposomes and various lipid suspensions (Deoyxcholic acid suspension, EYPC liposome, EYPC/deoxycholic acid micelle, DLPC liposome, and DLPC/deoxycholic acid micelle) was evaluated using the resonance energy transfer between NBD-PE and Rh-PE on EYPC liposomes. EYPC liposomes containing NBD-PE and Rh-PE (final concentration of lipid 6.6 μM) were mixed with various lipid suspensions (final concentration of lipid 6.6 μM) in 25 mM MES and 125 mM NaCl solution of varying pHs (4.5–7.4) and fluorescence intensities of NBD-PE and Rh-PE followed. Lipid mixing was followed by monitoring the fluorescence intensity ratio of NBD-PE to Rh-PE. The excitation wavelength of NBD-PE was 450 nm. The monitoring wavelengths for NBD-PE and Rh-PE were, respectively, 530 and 580 nm. To achieve complete lipid mixing, samples were dissolved in methanol, dried by evaporation, and resuspended in water.

### 2.7. Cellular Association of Micelles

The DC2.4 cells (5 × 10^4^ cells) cultured for three days in 24-well plates were washed with Hank’s balanced salt solution (HBSS, Sigma); then they were incubated in serum-free RPMI medium (500 μL). Micelles (25 μL) containing Rh-PE (0.6 mol %) were added gently to the cells and were incubated for 5 h at 4 or 37 °C. After incubation, the cells were washed three times with HBSS. The fluorescence intensity of these cells was found using a flow cytometer (Coulter Epics XL; Coulter Corp., Brea, CA, USA). For inhibition assay, cells were pre-treated with each inhibitor (chlorpromazine hydrochloride: 6.25–25 μg/mL [29], filipin: 2.5–10 μg/mL [29], amiloride: 2.5–10 mM [30]) for 30 min. After washing twice, micelles were added to these cells.

### 2.8. Detection of Intracellular Lipid Mixing

The DC2.4 cells (1.5 × 10^5^ cells) cultured for three days in 35-mm glass-bottom dishes were washed with HBSS; they were then incubated in serum-free RPMI medium (2 mL). Then, the liposomes or micelles (100 μL) containing NBD-PE and Rh-PE (each 0.6 mol %) were added gently to the medium of the cells and were incubated for 5 h at 37 °C. After incubation, the cells were washed with HBSS three times and were analyzed using confocal laser scanning microscopy (CLSM, LSM 5 EXCITER; Carl Zeiss Co. Inc., Oberkochen, Germany). Fluorescence of NBD-PE and Rh-PE was observed through specific pass filters (λ_em_ = 500–530 nm for NBD-PE and λ_em_ > 560 nm for Rh-PE) with excitation at 488 nm.

### 2.9. Cytoplasmic Delivery of Antigenic Proteins

The DC2.4 cells (1.5 × 10^5^ cells) cultured for 3 days in 35-mm glass-bottom dishes were washed with HBSS; then they were incubated in serum-free RPMI medium (2 mL). The FITC-OVA (50 μg) with or without DLPC liposomes or DLPC/deoxycholic acid micelles (100 μL) were added gently to the cells and were incubated for 4 h at 37 °C. After incubation, the cells were washed with HBSS three times. CLSM analysis of these cells was performed. Intracellular acidic compartments were stained with LysoTracker Red according to the manufacturer’s instructions.

### 2.10. Animals

Female C57BL/6 mice (H-2^b^, six weeks old) were purchased from Japan SLC, Inc. (Shizuoka, Japan). The experiments were conducted in accordance with the guidelines for animal experimentation of Terumo Corp.

### 2.11. ELIspot Assay

C57BL/6 mice were immunized intradermally with 16 μg of OVA, a mixture of OVA and CpG-ODN (2 μg), or a mixture of OVA, CpG-ODN and DLPC/deoxycholic acid micelles (1 μmol/mL, 2 μL) (total volume: 20 μL/mouse). After seven days, splenocytes were collected from each group. Splenocytes (2 × 10^6^ cells/well) were stimulated in vitro with 20 μg/mL of OVA peptide (SIINFEKL) or were left unstimulated (negative controls) for 40 h in a 96-well plate coated with anti-murine IFN-γ mAb. The IFN-γ-producing cells in the splenocyte populations were measured using mouse IFN-γ ELISPOT set and AEC substrate set (BD Biosciences, San Diego, CA) according to the manufacturer’s instructions. The data were expressed as the mean spot forming units (SFU) per million cells ± standard error (S.E.).

### 2.12. Statistical Analysis

The Tukey-Kramer method using Microsoft Excel was employed in the statistical evaluation of the results in Figure 3.

## 3. Results

### 3.1. Characterization of DLPC/Deoxycholic Acid Micelles

After the mixture of DLPC and deoyxcholic acid was dispersed in dPBS, the particle morphology was observed using AFM. Figure 1 shows that spherical particles with size of around 10 nm were observed, which corresponds to narrow size distribution measured by dynamic light scattering (Supplementary Materials Figure S1, average size: 12 nm) and is consistent with a previous report [25]. These data also support the nano-size micelle formation between DLPC and deoxycholic acid. Zeta potential of micelles was –30 mV, whereas liposomes composed of only DLPC shows −2.3 mV of zeta potentials. These results indicate that surface of micelles are covered with carboxyl groups derived from deoxycholic acid [25].

**Figure 2:**
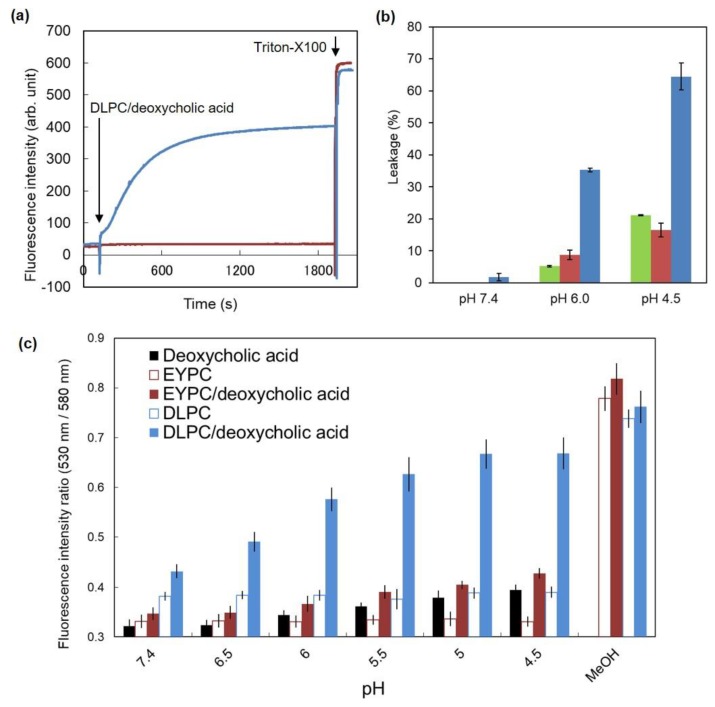
pH-responsive properties of DLPC/deoxycholic acid micelles. (**a**) Time courses of pyranine fluorescence for pyranine-loaded EYPC liposomes after addition of DLPC/deoxycholic acid micelles at pH 7.4 (red) or 5.0 (blue). Triton-X100 was added to dissociate the liposomal membrane completely. (**b**) Comparison of membrane disruptive activity for pyranine-loaded EYPC liposomes by addition of DOPE/CHEMS (green), DOPE/oleic acid (red) and DLPC/deoxycholic acid micelles (blue) at various pH. (**c**) Lipid mixing assay using EYPC liposomes containing Rh-PE/NBD-PE and various vehicles. Ratios of fluorescence intensity at 530 nm and 580 nm, which indicate a decrease of FRET by lipid mixing, are shown as a function of pH. Methanol was used to achieve complete lipid mixing.

Next, the pH-sensitivity of DLPC/deoxycholic acid micelles was examined. Fluorescence dye, pyranine-loaded EYPC liposomes were mixed with DLPC/deoxycholic acid micelles at pH 7.4 or 5.0. Leakage of pyranine was monitored (Figure 2a). At pH 7.4, no change was observed after the addition of DLPC/deoxycholic acid micelles, which indicates that DLPC/deoxycholic acid micelles are intact to lipid membranes at neutral pH. By contrast, the fluorescence intensity increased remarkably after addition of DLPC/deoxycholic acid micelles at pH 5.0 and reached a plateau within 20 min, suggesting that DLPC/deoxycholic acid micelles induced membrane disruption at acidic pH as reported in earlier reports of the relevant literature [25]. Membrane-disruptive performance of DLPC/deoxycholic acid micelles was compared with those of conventional pH-sensitive liposomes composed of DOPE/oleic acid or DOPE/CHEMS [26,27]. Figure 2b shows that DLPC/deoxycholic acid micelles induced pyranine leakage from EYPC liposomes more efficiently than DOPE/oleic acid or DOPE/CHEMS liposomes did, which indicates that DLPC/deoxycholic acid micelles have high membrane disruptive performance compared with conventional pH-sensitive liposomes.

Furthermore, membrane disruption mechanisms of DLPC/deoxycholic acid micelles were investigated using fluorescence resonance energy transfer (FRET). EYPC liposomes labeled with NBD-PE and Rh-PE were mixed with various lipid mixtures at various pH. Then the canceling of FRET by lipid mixing was measured (Figure 2c). In the cases of deoxycholic acid suspension, EYPC liposomes, EYPC/deoxycholic acid micelles, and DLPC liposomes, no change was observed for the fluorescence intensity ratios for NBD (530 nm) and Rh (580 nm) under excitation at 450 nm. These results indicate that these liposomes or lipid mixtures did not induce lipid mixing with EYPC liposomes under these experimental conditions. By contrast, DLPC/deoxycholic acid micelles exhibited a remarkable increase of fluorescence intensity ratio concomitantly with decreasing pH. These results suggest that lipid mixing with EYPC liposomes occurred at lower pH in the presence of DLPC/deoxycholic acid micelles, which leads to membrane disruption.

### 3.2. Interaction of DLPC/Deoxycholic Acid Micelles with Dendritic Cells

**Figure 3:**
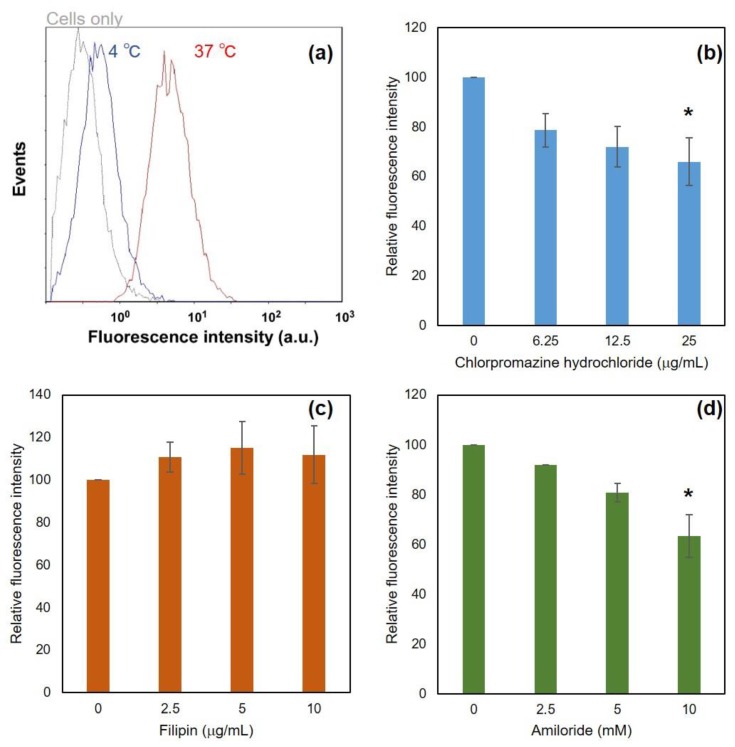
Interaction of DLPC/deoxycholic acid micelles with DC2.4 cells. (**a**) Effects of temperature for cellular association of micelles. DC2.4 cells were treated with Rh-PE-labeled micelles for 5 h at various temperatures. Cellular fluorescence was measured using a flow cytometer. (**b**–**d**) Effect of inhibitors for cellular association of micelles. Cellular association of Rh-PE-labeled micelles in the presence of various amounts of indicating inhibitors was evaluated. * *p* < 0.05 for the groups without inhibitor.

Interaction with DLPC/deoxycholic acid micelles with dendritic cells was investigated. Fluorescence-labeled DLPC/deoxycholic acid micelles were applied to DC2.4 cells and were incubated for 5 h. After washing, cellular fluorescence was measured using a flow cytometer (Figure 3a). At 37 °C, fluorescent distribution was significantly shifted to high fluorescence, whereas cellular fluorescence was almost equal to cell autofluorescence at 4 °C. These results indicate that DLPC/deoxycholic acid micelles were taken up by cells via energy-dependent endocytosis and showed little absorption on the cell surface. To elucidate endocytosis mechanisms, various inhibitors for endocytosis pathways were added before the addition of DLPC/deoxycholic acid micelles. Chlorpromazine and amiloride suppressed cellular association of DLPC/deoxycholic acid micelles dose-dependently (Figure 3b,d), whereas filipin had no affect (Figure 3c). Although filipin and amiloride hardly showed cytotoxicity to the cells under experimental conditions, chlorpromazine treatment decreased cell viability at high concentration (Supplementary Materials Figure S2). A decrease of cellular fluorescence intensity under chlorpromazine might be attributed by not only inhibition of clathrin-mediated endocytosis, but also its cytotoxicity. These results suggest that DLPC/deoxycholic acid micelles were taken up by dendritic cells mainly via macropinocytosis and slight clathrin-mediated endocytosis, but not caveola-mediated endocytosis.

### 3.3. Intracellular Behavior of DLPC/Deoxycholic Acid Micelles

**Figure 4:**
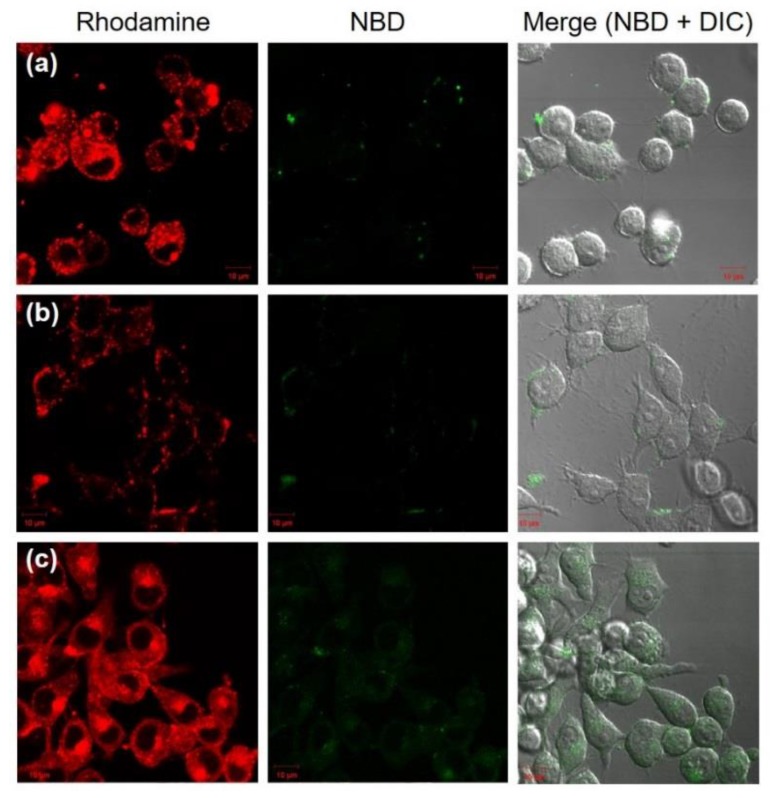
Confocal laser scanning microscopic (CLSM) images of DC2.4 cells treated with DLPC liposomes (**a**), EYPC/deoxycholic acid micelles (**b**) and DLPC/deoxycholic acid micelles (**c**) for 5 h. Fluorescence of NBD-PE and Rh-PE upon excitation at 488 nm was observed using a CLSM. Scale bar represents 10 μm.

As described in previous literature [23], when NBD is excited, NBD fluorescence is quenched by energy transfer to rhodamine on the same nanoparticles. In contrast, if nanoparticles induce the fusion or mixing with other membranes, NBD fluorescence becomes detectable as a result of the decrease of FRET efficiency even in the intracellular compartments [23]. Considering pH-sensitive properties of DLPC/deoxycholic acid micelles, intracellular behavior of micelles was investigated by detection of FRET canceling using confocal laser scanning microscopy (CLSM). DLPC liposomes, EYPC/deoxycholic acid micelles, and DLPC/deoxycholic acid micelles containing both NBD-PE and Rh-PE were applied to DC2.4 cells. Both Rh and NBD fluorescence within cells under excitation at 488 nm laser were detected using CLSM (Figure 4). In the cases of DLPC liposomes or EYPC/deoxycholic acid micelles, punctate red fluorescence was observed from the inside of cells or the cell surface, indicating that DLPC liposomes and EYPC/deoxycholic acid micelles were taken up by cells or absorbed onto the cell surface. However, slight green fluorescence was detected within cells. This result suggests that no lipid mixing of DLPC liposomes or EYPC/deoxycholic acid micelles with endo/lysosomal membranes occurred. By contrast, DLPC/deoxycholic acid micelles showed punctate and diffused fluorescence of rhodamine within cells. Furthermore, green fluorescence, which signifies the recovery of NBD fluorescence by lipid mixing, was observed clearly within cells (Figure 4c). These results indicate that DLPC/deoxycholic acid micelles induced lipid mixing with endo/lysosomes responding to weakly-acidic pH and that subsequent membrane rupture might lead the diffusion of Rh-PE molecules to inner spaces of cells.

**Figure 5:**
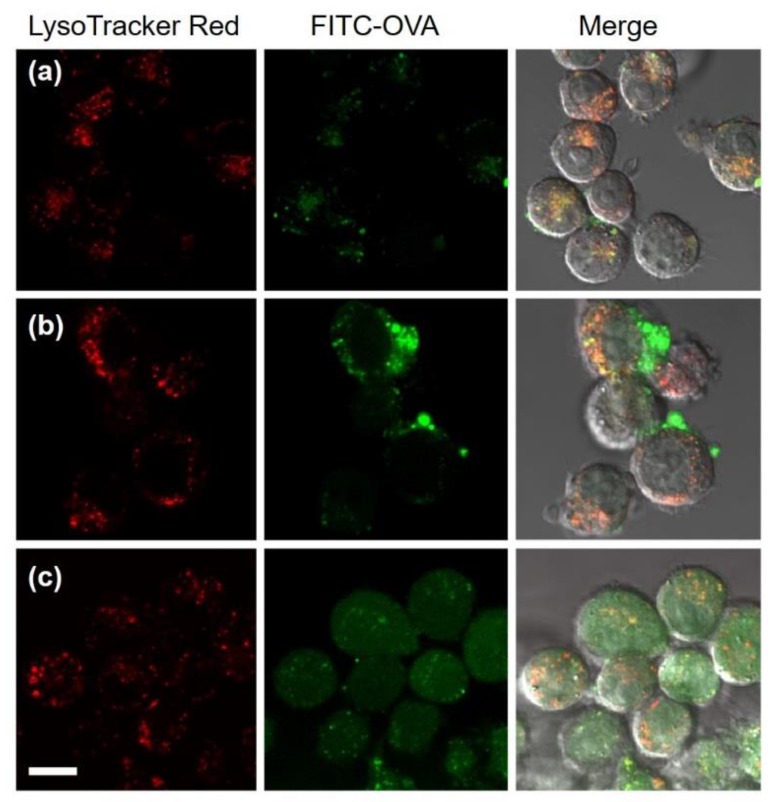
CLSM images of DC2.4 cells treated with FITC-OVA in the absence (**a**) or the presence of DLPC liposomes (**b**) or DLPC/deoxycholic acid micelles (**c**) for 5 h. Cells were also stained with LysoTracker Red. Scale bar represents 10 μm.

Intracellular delivery of model antigenic proteins using membrane-disruptive properties of DLPC/deoxycholic acid micelles was evaluated (Figure 5). FITC-labeled ovalbumin (FITC-OVA) was added to medium in the absence or presence of DLPC liposomes or DLPC/deoxycholic acid micelles. Intracellular distribution of FITC-OVA was observed. Free FITC-OVA fluorescence was overlapped with LysoTracker Red fluorescence, indicating that FITC-OVA was trapped in endo/lysosomes (Figure 5a). The presence of DLPC liposomes only slightly affected the intracellular distribution of FTIC-OVA (Figure 5b). By contrast, DLPC/deoxycholic acid micelles changed the location of FITC-OVA within cells considerably: FITC fluorescence diffused into whole cells (Figure 5c). These observations were also confirmed from the result of colocalization analysis (Supplementary Materials Figure S3). When DLPC/deoxycholic acid micelles and FITC-OVA molecules internalized to the same endo/lysosomes, membrane rupture by DLPC/deoxycholic acid micelles responding to acidic pH might promote the leakage of FITC-OVA molecules from these intracellular compartments into the cytosol.

### 3.4. Induction of Cellular Immunity In Vivo

**Figure 6:**
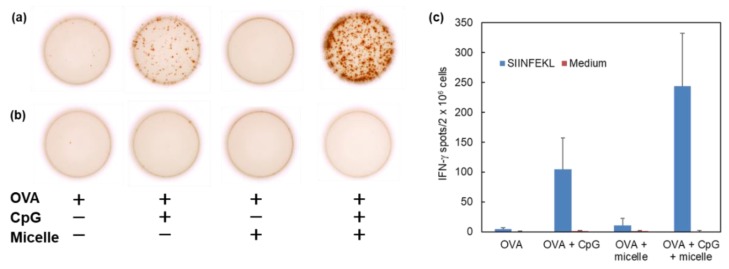
Induction of cellular immunity by pH-responsive micelles. C57BL/6 mice were immunized intradermally with 16 μg of OVA, OVA + CpG-ODN, or OVA + DLPC/deoxycholic acid micelle or OVA + CpG-ODN + DLPC/deoxycholic acid micelle. Splenocytes (2 × 10^6^ cells) were collected from the immunized mice on day 7 after immunization and were cultured with (**a**) or without (**b**) OVA CTL epitope peptide (SIINFEKL) for 40 h. (**a**,**b**) Typical micrographs of IFN-γ-producing spots measured by ELIspot. The number of IFN-γ-producing spots is shown (**c**).

To assess the cytoplasmic delivery performance of antigenic proteins by DLPC/deoxycholic acid micelles, the induction of antigen-specific cellular immunity in mice was examined. For induction of cellular immunity, not only cytoplasmic delivery of antigen, but also maturation of dendritic cells are crucially important. Here, CpG-ODN was combined with DLPC/deoxycholic acid micelles as an immunomodulator via toll-like receptor 9 (TLR9) [31,32]. Mice were intradermally immunized with OVA, CpG-ODN, and/or DLPC/deoxycholic acid micelles OVA-specific CTL response in spleen was evaluated using ELIspot assay (Figure 6). The addition of CpG-ODN slightly enhanced CTL induction, although quite strong CTL response was observed in the case of the combination of CpG-ODN and DLPC/deoxycholic acid micelles. However, no IFN-γ-producing CTL spot was detected in the absence of OVA epitope peptides during re-stimulation of splenocytes (Figure 6b,c). These results clearly demonstrate the efficient induction of OVA-specific CTL response in the spleen by administration of CpG-ODN and DLPC/deoxycholic acid micelles.

## 4. Discussion

Cytoplasmic delivery of antigen is crucial to induce antigen-specific CTL responses, which plays an important role in attacking tumor cells and eliminating virus-infected cells. Promotion of the cytoplasmic delivery of antigen using pH-responsive materials has been studied intensively, such as viral protein-incorporated liposomes, pH-sensitive liposomes, polymeric particles, and nanogels [10,11,24,33,34]. However, efficient vaccine delivery carriers with practical levels were only slightly reported. Here, we proposed an antigen-specific CTL induction system using a simple mixture of phospholipid and deoxycholic acid. Reportedly, a mixture of phospholipid with short alkyl chains (C12 or C10) and deoxycholic acid demonstrated pH-sensitive membrane disruptive ability [25]. Particularly, a combination of DLPC and deoxycholic acid exhibited pH-responsiveness in weakly-acidic pH regions corresponding to endo/lysosomal pH and formed nano-size micelles promoted intracellular delivery of macromolecules into macrophages [25]. As described in this paper, we specifically examined pH-responsive properties of DLPC/deoxycholic acid micelles and their application to CTL induction system.

Within 20 min, DLPC/deoxycholic acid micelles induced stronger membrane disruption at acidic pH (Figure 2a) than conventional pH-sensitive liposomes did (Figure 2b). Such a quick and strong pH-response might be beneficial to achieving membrane disruption of target membranes (endosomes or lysosomes) during the endocytic pathway and endosomal escape. At pH 4.5, almost complete lipid mixing was achieved by DLPC/deoxycholic acid micelles (Figure 2c), whereas leakage was 65% at the same pH (Figure 2b). This result suggests that DLPC/deoxycholic acid micelles first induce lipid mixing with the target membrane when micelles approach the target membrane because of high fluidity and flip-flop activity of DLPC [35]. Mixing of DLPC to the target membrane might decrease packing of the bilayer structure and increase the membrane permeability, causing a leakage of contents.

DLPC/deoxycholic acid micelles were taken up by dendritic cells mainly via macropinocytosis (Figure 3d) with little absorption in the cells (Figure 3a). This result might be attributable to the negatively charged surface of DLPC/deoxycholic acid micelles (‒30 mV) and low interaction with the cellular membrane at neutral pH. EYPC/deoxycholic acid micelles, which has almost the same size and surface properties with DLPC/deoxycholic acid micelles, showed relatively low cellular association compared with DLPC/deoxycholic acid micelles (Figure 4b,c). This might be attributed by less fluidity of EYPC molecules in micelles than DLPC, which further suppresses the interaction with the cellular membrane. After internalization to the cells, micelles induced lipid mixing even inside of the cells (Figure 4c). However, EYPC/deoxycholic acid micelles exhibited no pH-responsive membrane disruption (Figure 2c) and intracellular lipid mixing (Figure 4b), which also supports the importance of DLPC for induction of lipid mixing at acidic pH.

The presence of DLPC/deoxycholic acid micelles completely changed the intracellular fate of antigenic proteins internalized to cells along with micelles (Figure 5c). FITC-OVA molecules were delivered to cytosol of dendritic cells in the presence of DLPC/deoxycholic acid micelles. Lipid mixing of DLPC/deoxycholic acid micelles with endosomes might promote leakage of the FITC-OVA molecules to cytosol. Furthermore, lipid mixing and cytoplasmic delivery of FITC-OVA were observed even within 1 h (Supplementary Materials Figure S4), which reflect immediate pH-responsiveness of DLPC/deoxycholic acid micelles (Figure 2). Considering the cytoplasmic delivery mechanism by DLPC/deoxycholic acid micelles, both micelles and antigenic proteins should exist in the same endo/lysosome when endo/lysosomes are destabilized. Reportedly, endosomes and lysosomes are formed by the fusion of numerous endocytic vesicles during endocytic processes [36]. For example, gold nanoparticles were first taken up into endosomes as single particles and were then accumulated into the same endocytic vesicles during endocytic transportation [37]. Additionally, DLPC/deoxycholic acid micelles and FITC-OVA molecules might be taken up by cells independently, but micelles and FITC-OVA molecules might be sorted to the same endo/lysosomes during the endocytosis process, leading to cytoplasmic delivery of FITC-OVA by the pH-response of the micelles.

As expected, cytoplasmic delivery of antigen induced OVA-specific immune response in mice (Figure 6). By contrast, DLPC/deoxycholic acid micelles did not induce CTL response without CpG-ODN (Figure 6). Actually, addition of DLPC/deoxycholic acid micelles to dendritic cells hardly induced maturation of the dendritic cells even in high concentration (Supplementary Materials Figure S5). Such a property might be attributed to the weak interaction of micelles with the cellular membrane because of their negatively-charged surface. In addition, intradermally-injected micelles reached regional lymph node within 30 min and disappeared from injected site for 4 h (Supplementary Materials Figure S6). Micelles might be taken up by dermal dendritic cells and these dendritic cells might migrate to the lymph nodes. Another possibility is the direct transfer of micelles to lymph nodes because of its quite small particle size. After migration to the lymph nodes, DLPC/deoxycholic acid micelles delivered OVA into cytosol of dendritic cells, leading to induction of OVA-specific CTL responses. These results suggest that DLPC/deoxycholic acid micelles act as an “enhancer” of transfer to lymph node and cytoplasmic antigen delivery. Such characteristics of DLPC/deoxycholic acid micelles might be beneficial as additives of conventional or commercially-available vaccines because the DLPC/deoxycholic acid micelle, itself, does not induce any kind of immune response and does not disturb the intrinsic performance of vaccines. In addition, deoxycholic acid has already been approved by the FDA as an active principal of injectable drugs. DLPC also has a history of clinical use [38,39,40]. Therefore, pH-responsive DLPC/deoxycholic acid micelles are promising as additives of commercially-available vaccines to enhance cross-presentation of antigens for antigen-specific cellular immunity.

## 5. Conclusions

In this study, cytoplasmic delivery of antigen to dendritic cells was achieved by DLPC/deoxycholic acid micelles. A mixture of DLPC and deoxycholic acid formed spherical micelles with a size of 12 nm. DLPC/deoxycholic acid micelles exhibited lipid mixing with other lipid membranes at weakly-acidic pH, which led to remarkable leakage from liposomes or endosomal escape of co-existing antigenic proteins after internalization to dendritic cells. The DLPC/deoxycholic acid micelle itself hardly induced maturation of dendritic cells and the combination with adjuvants promoted the induction of antigen-specific cellular immune responses after intradermal injection in mice. Therefore, DLPC/deoxycholic acid micelle is promising delivery platform for cytoplasmic delivery and CTL-inducing system to achieve cancer immunotherapy or efficient treatment of infectious diseases.

## Supplementary Materials

The following are available online at www.mdpi.com/2076-393X/5/4/41/s1; Figure S1: Size distribution of DLPC/deoxycholic acid micelle, Table S1: Compositions of all formulations used in this study, Figure S2: Cell viability of DC2.4 cells treated with endocytic inhibitors, Figure S3: Colocalization analysis for Figure 5: Figure S4: Intracellular distribution of FITC-OVA with DLPC/deoxycholic acid micelles after 1 h, Figure S5: Effects of micelle treatment on maturation markers on spleen-derived dendritic cells, Figure S6: Tracking of micelles after intradermal injection.

## References

[1] Sridhar S., Begom S., Bermingham A., Hoschler K., Adamson W., Carman W., Bean T., Barclay W., Deeks J.J., Lalvani A. (2013). Cellular immune correlates of protection against symptomatic pandemic influenza. Nat. Med..

[2] Sandgren K.J., Bertram K., Cunningham A.L. (2016). Understanding natural herpes simplex virus immunity to inform next-generation vaccine design. Clin. Transl. Immunol..

[3] Holz L.E., Fernandez-Ruiz D., Heath W.R. (2016). Protective immunity to liver-stage malaria. Clin. Transl. Immunol..

[4] Yang S., Gattinoni L., Liu F., Ji Y., Yu Z., Restifo N.P., Rosenberg S.A., Morgan R.A. (2011). In vitro generated anti-tumor T lymphocytes exhibit distinct subsets mimicking in vivo antigen-experienced cells. Cancer Immunol. Immunother..

[5] Banchereau J., Steinman R.M. (1998). Dendritic cells and the control of immunity. Nature.

[6] Mellman I., Steinman R.M. (2001). Dendritic cells: Specialized and regulated antigen processing machines. Cell.

[7] Joffre O.P., Segura E., Savina A., Amigorena S. (2012). Cross-presentation by dendritic cells. Nat. Rev. Immunol..

[8] Garulli B., Di Mario G., Sciaraffia E., Accapezzato D., Barnaba V., Castrucci M.R. (2013). Enhancement of T cell-mediated immune responses to whole inactivated influenza virus by chloroquine treatment in vivo. Vaccine.

[9] Chen J., Li Z., Huang H., Yang Y., Ding Q., Mai J., Guo W., Xu Y. (2011). Improved antigen cross-presentation by polyethyleneimine-based nanoparticles. Int. J. Nanomed..

[10] Bungener L., Serre K., Bijl L., Leserman L., Wilschut J., Daemen T., Machy P. (2002). Virosome-mediated delivery of protein antigens to dendritic cells. Vaccine.

[11] Kunisawa J., Nakagawa S., Mayumi T. (2001). Pharmacotherapy by intracellular delivery of drugs using fusogenic liposomes: Application to vaccine development. Adv. Drug Deliv. Rev..

[12] Seki K., Tirrell D.A. (1984). pH-Dependent complexation of poly(acrylic acid) derivatives with phospholipid vesicle membrane. Macromolecules.

[13] Murthy N., Robichaud J.R., Tirrell D.A., Stayton P.S., Hoffman A.S. (1999). The design and synthesis of polymers for eukaryotic membrane disruption. J. Control. Release.

[14] Lackey C.A., Murthy N., Press O.W., Tirrell D.A., Hoffman A.S., Stayton P.S. (1999). Hemolytic activity of pH-responsive polymer-streptavidin bioconjugates. Bioconj. Chem..

[15] Flanary S., Hoffman A.S., Stayton P.S. (2009). Antigen delivery with poly(propylacrylic acid) conjugation enhances MHC-1 presentation and T-cell activation. Bioconj. Chem..

[16] Foster S., Duval C.L., Crownover E.F., Hoffman A.S., Stayton P.S. (2010). Intracellular delivery of a protein antigen with an endosomal-releasing polymer enhances CD8 T-cell production and prophylactic vaccine efficacy. Bioconj. Chem..

[17] Harada A., Teranishi R., Yuba E., Kono K. (2017). Effect of the side chain spacer structure on the pH-responsive properties of polycarboxylates. J. Biomater. Sci. Polym. Ed..

[18] Kono K., Igawa T., Takagishi T. (1997). Cytoplasmic delivery of calcein mediated by liposomes modified with a pH-sensitive poly(ethylene glycol) derivative. Biochim. Biophys. Acta.

[19] Sakaguchi N., Kojima C., Harada A., Kono K. (2008). Preparation of pH-sensitive poly(glycidol) derivatives with varying hydrophobicities: Their ability to sensitize stable liposomes to pH. Bioconj. Chem..

[20] Yuba E., Tajima N., Yoshizaki Y., Harada A., Hayashi H., Kono K. (2014). Dextran derivative-based pH-sensitive liposomes for cancer immunotherapy. Biomaterials.

[21] Yuba E., Yamaguchi A., Yoshizaki Y., Harada A., Kono K. (2017). Bioactive polysaccharide-based pH-sensitive polymers for cytoplasmic delivery of antigen and activation of antigen-specific immunity. Biomaterials.

[22] Yuba E., Uesugi S., Miyazaki M., Kado Y., Harada A., Kono K. (2017). Development of pH-sensitive dextran derivatives with strong adjuvant function and their application to antigen delivery. Membranes.

[23] Yuba E., Harada A., Sakanishi Y., Kono K. (2011). Carboxylated hyperbranched poly(glycidol)s for preparation of pH-sensitive liposomes. J. Control. Release.

[24] Yuba E., Harada A., Sakanishi Y., Watarai S., Kono K. (2013). A liposome-based antigen delivery system using pH-sensitive fusogenic polymers for cancer immunotherapy. Biomaterials.

[25] Miyamoto N., Fujii S., Mochizuki S., Sakurai K., Sakaguchi N., Koiwai K. (2017). A two-component micelle with emergent pH responsiveness by mixing dilauroyl phosphocholine and deoxycholic acid and its delivery of proteins into the cytosol. Colloids Surf. B Biointerfaces.

[26] Hafez I.M., Cullis P.R. (2000). Cholesteryl hemisuccinate exhibits pH sensitive polymorphic phase behavior. Biochim. Biophys. Acta.

[27] Liu D., Huang L. (1989). Role of cholesterol in the stability of pH-sensitive, large unilamellar liposomes prepared by the detergent-dialysis method. Biochim. Biophys. Acta.

[28] Shen Z., Reznikoff G., Dranoff G., Rock K.L. (1997). Cloned dendritic cells can present exogenous antigens on both MHC class I and class II molecules. J. Immunol..

[29] Yamada M., Oeda A., Jung J., Iijima M., Yoshimoto N., Niimi T., Jeong S.Y., Choi E.K., Tanizawa K., Kuroda S (2012). Hepatitis B virus envelope L protein-derived bio-nanocapsules: Mechanisms of cellular attachment and entry into human hepatic cells. J. Control. Release.

[30] Khalil I.A., Kogure K., Futaki S., Harashima H. (2006). High density of octaarginine stimulates macropinocytosis leading to efficient intracellular trafficking for gene expression. J. Biol. Chem..

[31] Bode C., Zhao G., Steinhagen F., Kinjo T., Klinman D.M. (2011). CpG DNA as a vaccine adjuvant. Expert Rev. Vaccines.

[32] Kawai T., Akira S. (2010). The role of pattern-recognition receptors in innate immunity: Update on Toll-like receptors. Nat. Immunol..

[33] Bachelder E.M., Beaudette T.T., Broaders K.E., Fréchet J.M.J., Albrecht M.T., Mateczun A.J., Ainslie K.M., Pesce J.T., Keane-Myers A.M. (2010). In vitro analysis of acetalated dextran microparticles as a potent delivery platform for vaccine adjuvants. Mol. Pharm..

[34] Tahara Y., Akiyoshi K. (2015). Current advances in self-assembled nanogel delivery systems for immunotherapy. Adv. Drug Deliv. Rev..

[35] Sapay N., Bennett W.F.D., Tieleman D.P. (2009). Thermodynamics of flip-flop and desorption for a systematic series of phosphatidylcholine lipids. Soft Matter.

[36] Huotari J., Helenius A. (2011). Endosome maturation. EMBO J..

[37] Liu M., Li Q., Liang L., Li J., Wang K., Li J., Lv M., Chen N., Song H., Lee J. (2017). Real-time visualization of clustering and intracellular transport of gold nanoparticles by correlative imaging. Nat. Commun..

[38] Lee J.M., Lee Y.K., Mamrosh J.L., Busby S.A., Griffin P.R., Pathak M.C., Ortlund E.A., Moore D.D. (2011). A nuclear-receptor-dependent phosphatidylcholine pathway with antidiabetic effects. Nature.

[39] Ganley D.J., Wilt J. (2012). Methods for the Synthesis and Purification of Deoxycholic Acid.

[40] Saari M., Vidgren M.T., Koskinen M.O., Turjanmaa V.M., Nieminen M.M. (1999). Pulmonary distribution and clearance of two beclomethasone liposome formulations in healthy volunteers. Int. J. Plast..

