# Supplementary material for: pH-Responsive Micelle-Based Cytoplasmic Delivery System for Induction of Cellular Immunity
Source: Vaccines (Basel). 2017 Nov 4;5(4):41. doi: 10.3390/vaccines5040041 (PMC5748608; doi:10.3390/vaccines5040041)
Supplement: Supplementary file 1 [file vaccines-05-00041-s001.pdf]

Supplementary Materials

pH-Responsive Micelle-Based Cytoplasmic Delivery System for Induction of Cellular Immunity

Eiji Yuba [1,*], Naoki Sakaguchi [2], Yuhei Kanda [1], Maiko Miyazaki [1] and Kazunori Koiwai [2]

[1] Department of Applied Chemistry, Graduate School of Engineering, Osaka Prefecture University, 1–1 Gakuen–cho, Naka–ku, Sakai, Osaka 5998531, Japan; kndyh.june8@gmail.com (Y.K.); su108056@edu.osakafu-u.ac.jp (M.M.)

[2] Terumo Corp., Ashigarakami–gun, Kanagawa 2590151, Japan; Naoki_Sakaguchi@terumo.co.jp (N.S.); tgwmt240@jcom.zaq.ne.jp (K.K.)

* Correspondence: yuba@chem.osakafu-u.ac.jp; Tel.: +81-72-254-9913; Fax: +81-72-254-9330

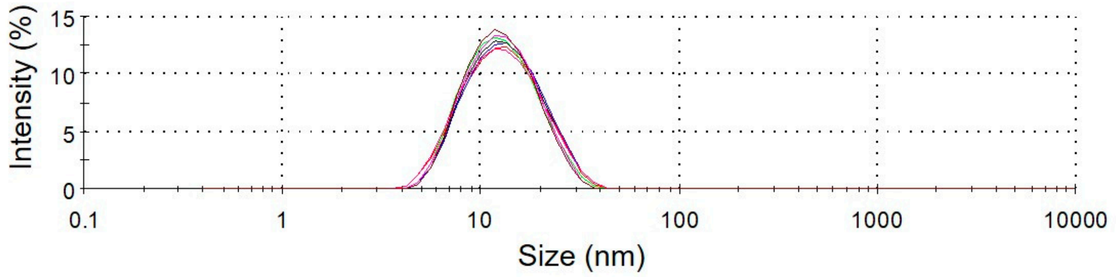

Figure S1. Size distribution of DLPC/deoxycholic acid micelles measured in 1.0 mM HEPES buffer (pH 7.4) using a Zetasizer Nano ZS ZS90 (Malvern Instruments Ltd, Worcestershire, UK).

Table S1. Composition of micelles or liposomes used in this study.

Micelle/liposomes	Compositions (mol%)
DLPC/DA micelle	DLPC/DA = 1/1.6
DA suspension	DA only
EYPC liposome	EYPC only
EYPC/DA micelle	EYPC/DA = 1/1.6
DLPC liposome	DLPC only
DOPE/CHEMS liposome	DOPE/CHEMS = 3/2
DOPE/oleic acid liposome	DOPE/oleic acid = 7/3
Rh-labeled DLPC/DA micelle	DLPC/DA/Rh-PE = 1/1.6/0.006
NBD/Rh-labeled DLPC liposome	DLPC/Rh-PE/NBD-PE = 1/0.006/0.006
NBD/Rh-labeled EYPC/DA micelle	EYPC/DA/Rh-PE/NBD-PE = 1/1.6/0.006/0.006
NBD/Rh-labeled DLPC/DA micelle	DLPC/DA/Rh-PE/NBD-PE = 1/1.6/0.006/0.006
Pyranine-loaded EYPC liposome	EYPC only, encapsulating pyranine/DPX

DA: Deoxycholic acid.

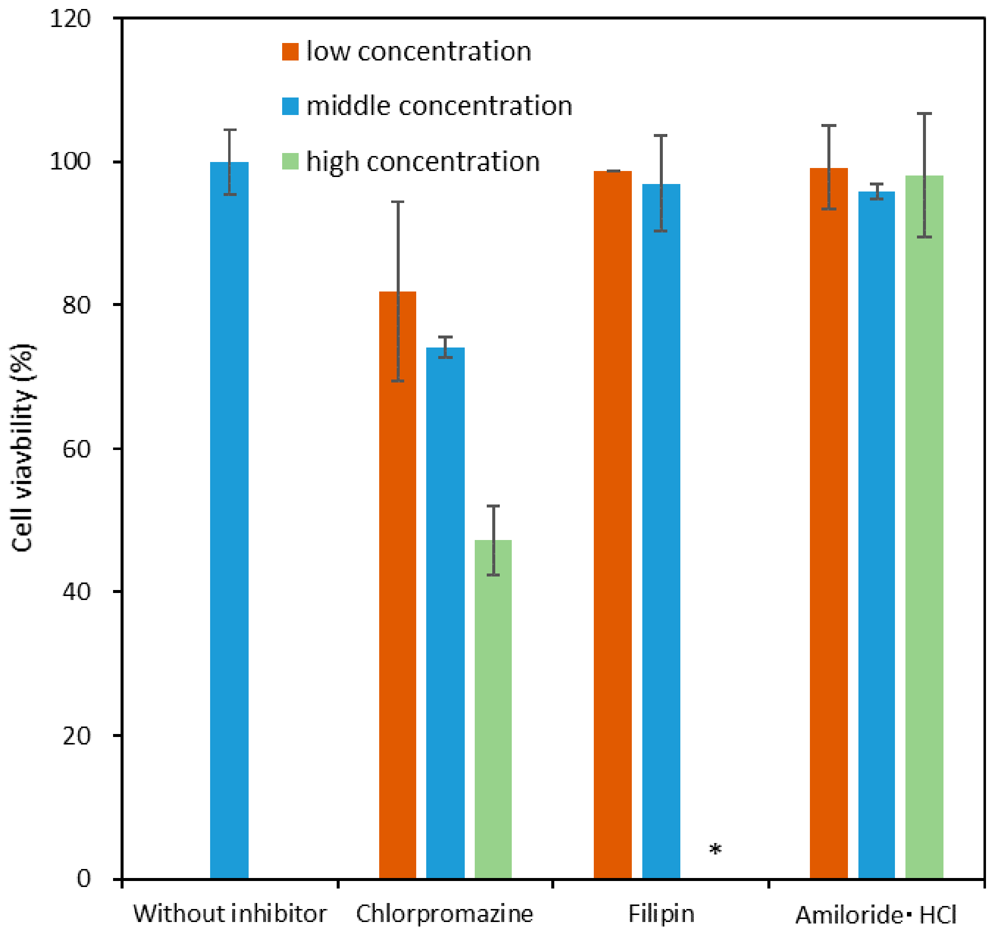

Figure S2. Cell viability of DC2.4 cells treated with chlorpromazine (6.25, 12.5, 25 g/mL), filipin (2.5, 5 g/mL), or amiloride (2.5, 5, 10 mM) for 30 min and subsequent 5 h culture in the absence of FBS. Cell viability was measured using Cell Count Reagent SF (Nacalai tesque).* Not tested.

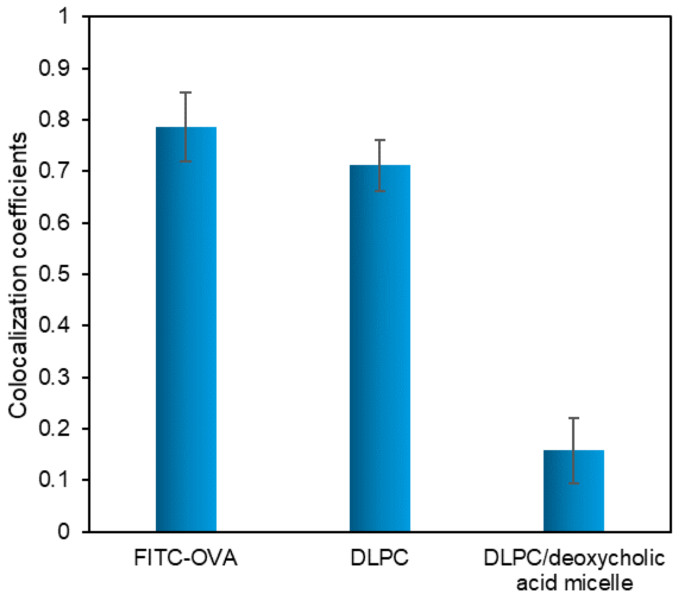

Figure S3. Colocalization of FITC fluorescence derived from FITC-OVA with LysoTracker Red fluorescence calculated from CLSM images in Figure 5. More than 50 cells in each groups were evaluated. Colocalization coefficients were calculated from (green pixels that overlaps with red pixels) / (total green pixels) in each cell of interest.

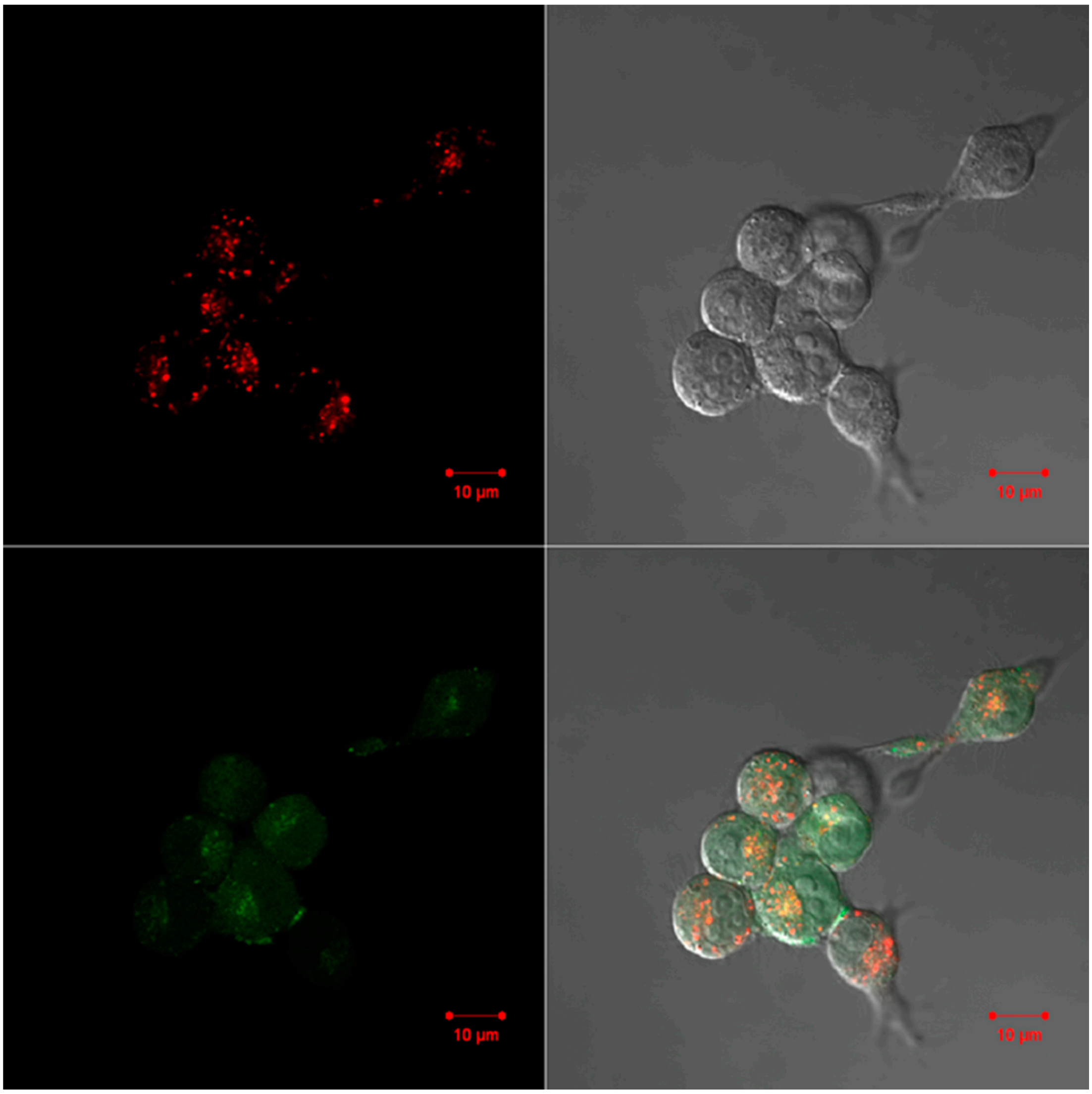

Figure S4. CLSM images of DC2.4 cells treated with FITC-OVA (green) in the presence of DLPC/deoxycholic acid micelles for 1 h. Cells were also stained with LysoTracker Red (red). Scale bar represents 10 μm.

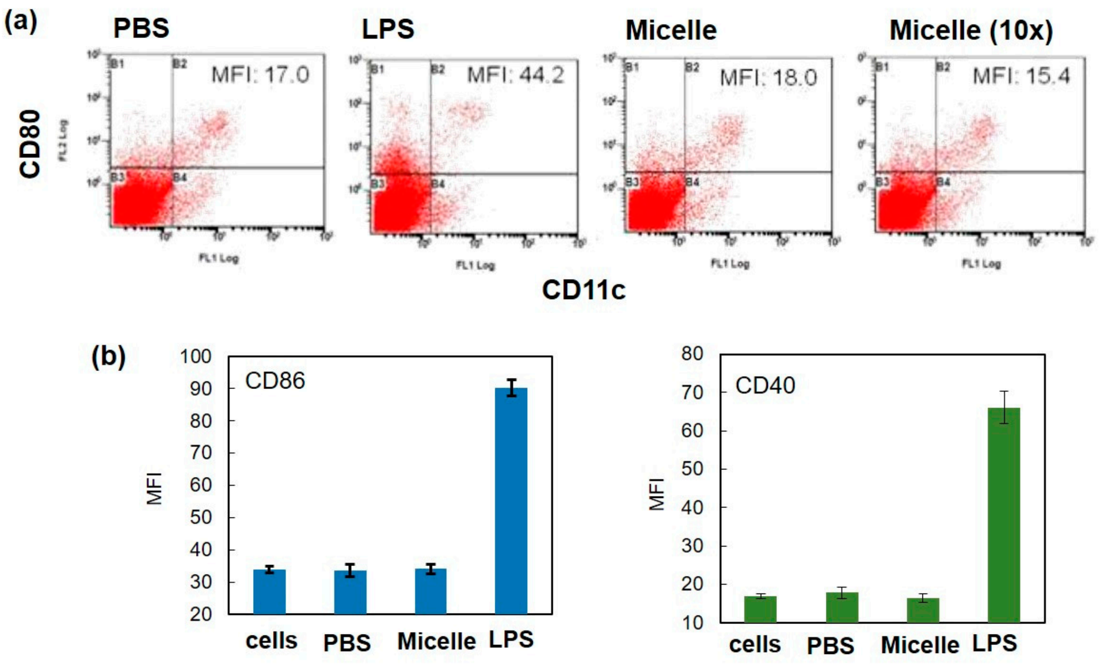

Figure S5. DLPC/deoxycholic acid micelles showed no stimulation to dendritic cells. Mouse splenocytes were treated with lipopolysaccharide (LPS, 500 ng/mL) or DLPC/deoxycholic acid micelles (5 nmol/mL or 50 nmol/mL) overnight. After washing, cells were double-stained using FITC-labeled CD11c antibody, PE-labeled CD80/CD86/CD40 antibodies according to the manufacture's instruction (eBioscience). After washing, flow cytometrical analysis was performed. (**a**) Typical results of cells stained by CD80/CD11c. (**b**) CD80/CD40 expression in the cells.

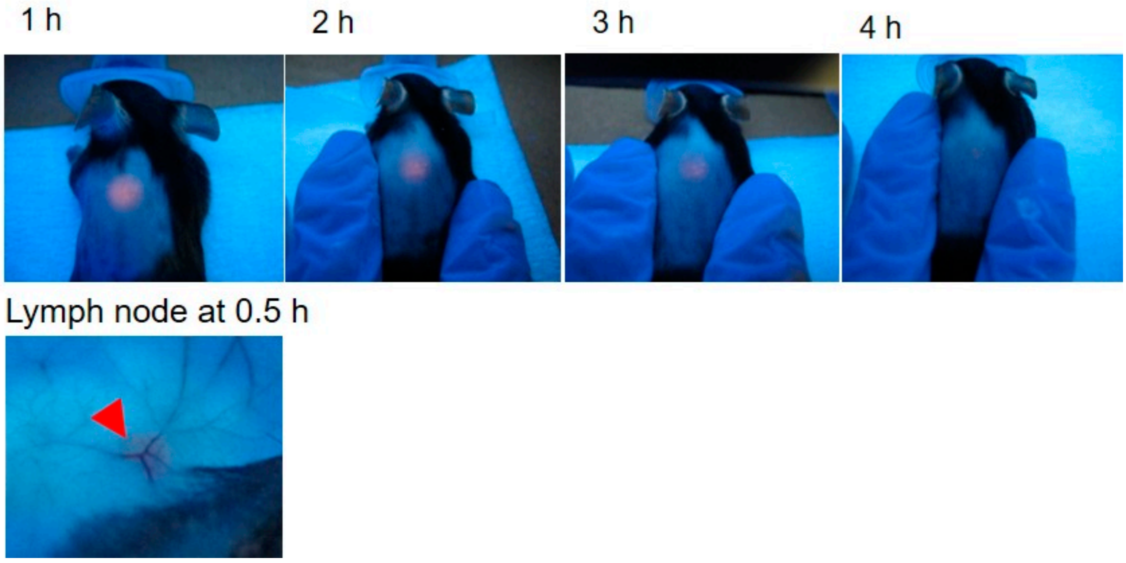

Figure S6. Fluorescently-labeled DLPC/deoxycholic acid micelles were intradermally injected into mice. Then, the location of micelles was detected under UV light irradiation at various time points. At 0.5 h, the regional lymph node (arrowhead) was also observed.
